# The Impact of Selected ESD Parameters on the Properties of Tungsten Layers

**DOI:** 10.3390/ma18194581

**Published:** 2025-10-02

**Authors:** Piotr Młynarczyk, Damian Bańkowski, Wojciech Depczyński

**Affiliations:** Department of Materials Science and Materials Technology, Faculty of Machatronics and Mechanical Engineering, Kielce University of Technology, al. 1000-lecia P.P. 7, 25-314 Kielce, Poland; dbankowski@tu.kielce.pl (D.B.); wdep@tu.kielce.pl (W.D.)

**Keywords:** ESD, microscopic examination, tribological tests, W-Ni-Co layer

## Abstract

This article presents studies of surface layers produced by electro-spark deposition (ESD) on cast iron using a W-Ni-Co sintered electrode. To minimize the number of required experiments, a two-factor, five-level Hartley experimental design was chosen. The assessment involved observing the effect of voltage and capacitor capacity during the ESD process (on layer thickness and wear of the sample and counter-sample under technically dry friction conditions). Microscopic and tomographic observations were performed to analyze the thickness and structure of the layers. Image analysis methods were employed to examine the cross-section of the layers. ESD diffusion analyses were performed on the produced layer. Scanning electron microscopy (SEM) and energy dispersive X-ray spectroscopy (EDX) were performed to characterize the microstructure and composition of the coating. In addition, in order to evaluate the performance properties of tungsten coatings, the tribological tests were also conducted on a TRB^3^ Ball-on-Disc testing device. Hardness tests confirm an increase in the hardness of cast iron with a tungsten layer by over 400 µHV. The tests showed that higher voltages during the ESD process result in thicker layers and reduced wear of the sample with a tungsten layer at the expense of increased wear of the counter-sample (ball).

## 1. Introduction

**Figure 1:**
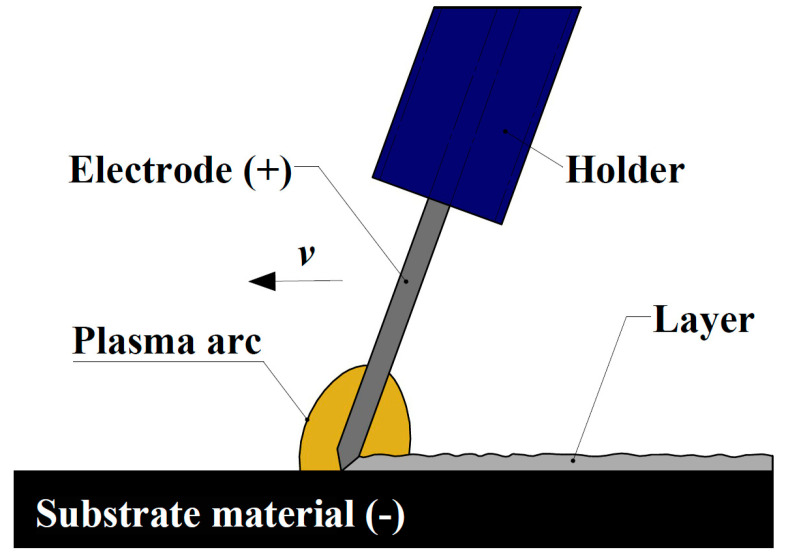
Schematic of surface layer forming by electro-spark deposition method.

Electric discharge deposition is a pulsed-arc microwelding process that uses short-duration, high-current electrical pulses to weld consumable electrode material to a metallic substrate. The schematic of surface layer forming by electro-spark deposition method is presented in Figure 1. It is distinguished from other arc welding processes by the fact that the spark duration is limited to a few microseconds and the spark frequency is 1000 Hz or less. ESD offers particular advantages when coating or repairing materials considered difficult to weld due to heat-affected zone (HAZ) issues. Furthermore, many materials can be processed without undergoing heat treatment before or after ESD.

The electro-spark deposition process utilizes microsecond electrical pulses to induce local melting and rapid solidification, enabling the incremental transfer of electrode material onto the substrate surface [1,2]. Spark erosion has many applications in the restoration and coating of electrically conductive materials. Due to the very low heat input, it is ideal for use in regenerating and coating metals susceptible to the influence of the heat-affected zone (HAZ), such as H13 casting moulds [3]. Spark erosion is a surface treatment technology in which a pulsating micro-arc generated between the electrode and the substrate ionizes the air, creating a high temperature in a small area where alloying occurs. This creates a small heat-affected zone, as well as a composite coating with a high melting point generated by a temporary increase in temperature of several thousand degrees [4,5]. Spark erosion coatings are considered wear- and corrosion-resistant and can be used for elements of ship propellers, casting moulds, fuel supply system components, exhaust system components, gas turbine components, and cannon cradles. For example, critical areas of casting matrix surfaces may be damaged by melting and thermal fatigue [6,7]. In these cases, applying protective or restorative coatings can increase the service life of foundry moulds. Another promising area of application for spark erosion deposition (ESD) is the treatment of titanium alloy surfaces for biomedical applications. These coatings should meet the following requirements: strong bond strength, good wear resistance, excellent anti-corrosion properties, bio-compatibility and high biological activity. However, most of the current technical methods published in the literature cannot perfectly meet these requirements. For example, the synthesis of bioactive ceramic coatings using procedures such as plasma spraying [8], physical vapour deposition [9] and electrodeposition [10], as well as anodic oxidation [11], often results in poor bond strength. Some surface strengthening technologies, such as high-temperature nitriding [12] and sintering of active ceramics [13], can damage the properties of the substrate. Another area of application for this technique is biomedical engineering.

Titanium alloys are currently the most attractive metallic materials for replacing hard tissues in biomedical applications due to their excellent mechanical, biological, and anti-corrosion properties [14]. However, their poor wear and corrosion resistance [15], adverse biological reactions [16,17,18], and other disadvantages limit their safe and reliable wide-scale application in clinical practice. The search for surface modification techniques, such as physical, chemical, and biological treatments, to improve wear resistance and increase biological activity, remains of great interest to scientists [19,20,21].

Widespread access to verified research results on ESD is leading to this technique being used for less demanding materials too. In [22], the ESD technique was used to coat grey iron (GI) with nickel (Ni), AISI 304 stainless steel (304 SS), and Inconel 718 superalloy. The microstructures of the coated ductile iron were examined in terms of microhardness, film thickness, and corrosion resistance. According to a review of the literature, no coating investigations employing the ESD technique on cast iron have been conducted. Previous research on ESD coatings has focused on improving the hardness and wear resistance of metallic materials [23].

As electro-spark coatings are resistant to wear and corrosion, they can be used for components such as the following:

- Ship propeller components [24];
- Casting moulds [25];
- Fuel supply system components;
- Exhaust system components;
- Gas turbine component [26];
- Cannon cradle [27].

## 2. Materials and Methods

### 2.1. Planned Experiment

Due to the limited number of experiments that could be carried out, a two-factor five-level Hartley experimental plan was selected.

Statistica 10 64-bit software was used to conduct the planned experiment. A five-level plan was chosen in order to examine the influence of independent variables on the output factors to a greater extent than a three-level plan [28]. The process variability ranges were selected for the following:

- Voltage—U, took the values 300, 400, 500, 600, 700 V, which correspond to the code values: −2; −1; 0; 1; 2;
- Capacitor capacity—f, took the values 25, 50, 75, 133 µF, which correspond to the code values: −2, −1, 0, 2 [29].

For standard plans, the core of the plan usually has a resolution of V (or higher). However, this is not necessary, and in cases where the experiments are expensive or where it is not necessary to perform a statistically robust model adequacy test, a plan with a resolution of III can be selected as the core [30]. For this reason, the Small Composition Plans method was chosen as an effective method that minimizes the number of systems tested.

If it is necessary to consider a large number of variables in order to identify those that may be important (i.e., affect the output), an experimental design can be used that allows the largest number of main effects of the input variables to be investigated with the smallest possible number of plan layouts. This involves constructing a design of experiments with a resolution of III with as few layouts as possible. One possible way to construct such a plan is to combine all interactions with ‘new’ main effects. These plans are often called saturated plans, as all information provided by such a plan is used to determine the parameters, and thus no degrees of freedom remain for estimating inaccuracies in the ANOVA analysis [30].

Hartley’s test (also known as the Fmax test or Hartley’s Fmax) is a statistical test used to test the homogeneity of variance [31]. The test is performed before ANOVA to check whether the assumption of equality of variance between two or more groups being compared is met (assuming that the samples analyzed come from a normally distributed population) [32]. The test was developed by German-American statistician Herman Otto Hartley. Table 1 presents the results of the mass loss of the sample and counter-sample after tribology test experiments, and the thickness of the obtained layers depending on the ESD process parameters.

The results of friction pair wear without a coating were as follows: the loss of sample mass after tribological testing was Δmw = 0.0075 g, and the loss of reference sample mass after tribological testing was Δmb = 0.0003 g.

### 2.2. Materials and Electro-Spark Deposition

**Figure 2:**
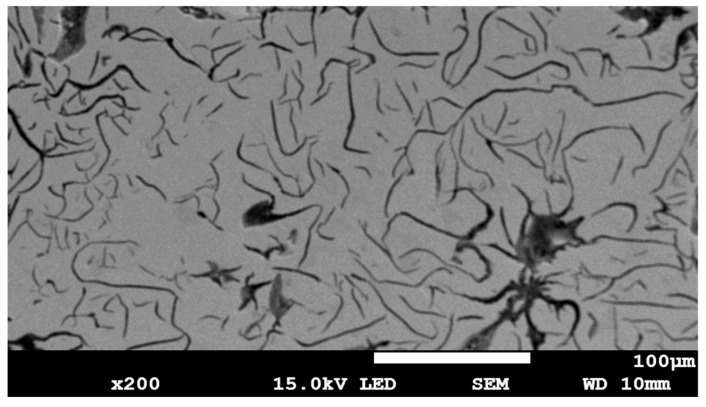
Scanning electron microscope (SEM) micrograph of the cast iron sample used in the experiment.

The material used in the study was a cast iron. X-ray fluorescence spectrometry (XRF): the GNR Optima TX 200 (GNR, Milan, Italy) was used to analyze the chemical composition of cast iron components. Table 2 provides information on the chemical composition of the cast iron used in the research experiments, and Figure 2 shows an SEM micrograph.

**Figure 3:**
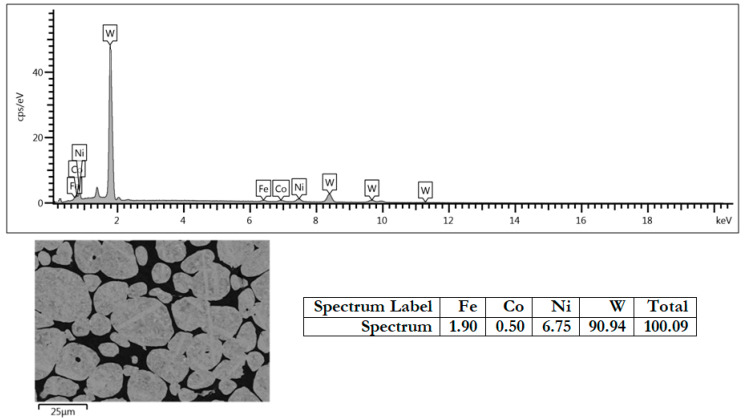
The material of the electrode used to create the layer by the ESD technique. Surface analysis in Weight %.

The chemical composition and morphology of the sample allow the material to be identified as grey cast iron on a ferritic matrix, whose metallic matrix consists mainly of ferrite. Test samples were taken from the worn bucket feeder. Rectangular samples measuring 10 × 15 mm, were prepared for metallography investigations, and round samples with a diameter of 40 mm for tribological tests. Next, the layers were applied using the ESD method. All ESD coatings were applied at a frequency of 50 Hz, with a voltage ranging from 300 to 700 V and a capacitance ranging from 25 to 133 μF. The ESD coatings were applied until the surface was uniformly coated, as judged by the operator. The coating is formed using a vibrating electrode attached to an applicator during the ESD process. The ESD coating consists of sparks produced when lightly touched by cast iron. Figure 3 shows the chemical analysis of the electrode used as the coating material.

The chemical analysis results show the chemical composition of the tungsten sinter used as an electrode for applying the wear-resistant layer. The micrograph accompanying the element distribution chart shows tungsten grains in a matrix of nickel, iron, and cobalt. The results are shown in raw (non-normalized) form, so the sum of the masses of the elements deviates from 100%, which is a classic problem in interpreting EDS results. In the case of non-normalized data, “raw” values are given, without forcing the sum to be 100%.

### 2.3. A Metallographic Study

**Figure 4:**
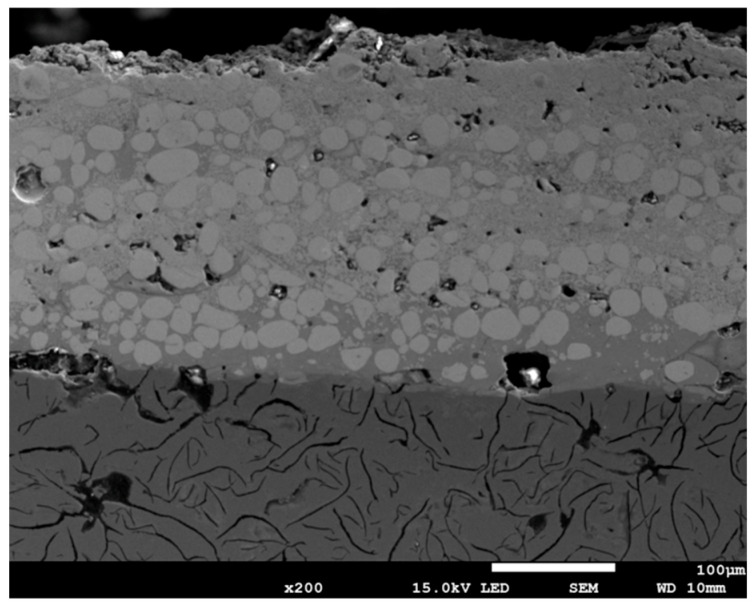
SEM micrograph of the produced surface layer 200×, parameters: voltage 700 V, 50 μF.

A metallographic inspection is being carried out on the substrate and the deposition material. Metallographic inspection sections have been cut from cross-sections of samples, with the material undergoing testing embedded in Technovit 5000 (Kulzer GmbH, Hanau, Germany) conductive resin. Subsequent grinding was carried out on a Presi Minitech 263 (Presi, Eybens, France) machine, and the material was then polished. The following SIC papers were used in the study: 230, 400, 600, 1200, and 2500. The final stage of the process involved polishing on a Struers LaboPol-5 (Struers, Copenhagen, Denmark) using 1 µm polycrystalline diamond suspension and SUPRA cloth (Microdiamant, Kempen, Germany). In the present study, the microstructures of the polished surfaces were exposed by means of 2% Nital complete etching. The OEM microscope, the Nikon Eclipse MA 200 (Nikon, Minato-ku, Japan), was utilized with the NIS 4.20-Elements Viewer imaging software version XT 6.8, while the SEM JEOL 7100F microscope (JEOL, Tokyo, Japan) was employed with an EDS (Oxford Instruments, Bucks, UK) probe. These instruments were used to study the microstructures and to acquire a microphotograph in BSE mode. The same optical microscope was employed for the measurement of layer thickness using an optical instrument micrometre. The SEM observations of the obtained layer are presented in Figure 4.

**Figure 5:**
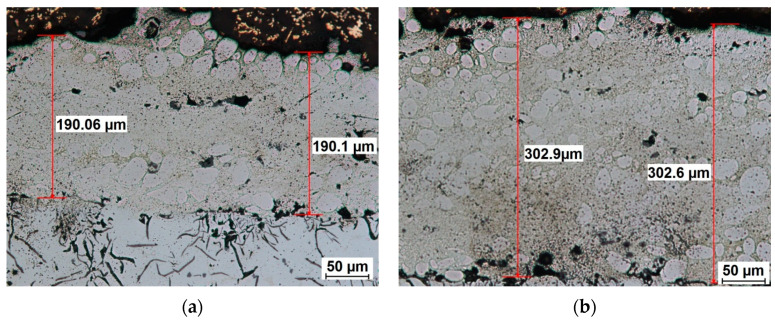
Microphotograph showing thickness of the obtained layer, 200×, parameters: (**a**) voltage 500 V, 133 μF, and (**b**) voltage 700 V, 75 μF.

The layer thickness was determined through the calculation of the mean of at least ten readings, obtained through duplicate measurements taken from five distinct locations on the metallographic sample surface. These readings were acquired using an OEM Nikon Eclipse MA 200 microscope. As illustrated in Figure 5, the thickness of the layers obtained using the ESD technique with different parameters is demonstrated.

### 2.4. Hardness Properties

**Figure 6:**
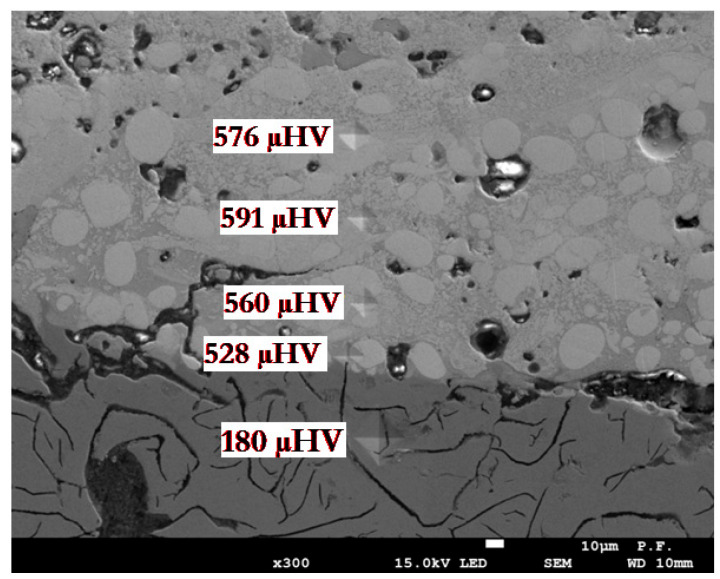
Traces of a Vickers indenter on the cross-section of the layer with marked microhardness values.

To measure the hardness of the coatings an Innovatest NEXUS 4000 (Innovatest, Maastricht, The Netherlands) Vickers hardness tester, weighing 100 g, was used. The hardness measurement results for all coated samples were very similar. Figure 6 shows a typical hardness distribution across the applied layer using the EDS technique.

### 2.5. Tribology Tests

Tribological tests were conducted on a TRB^3^ Tribometer (Anton Paar, Baden, Switzerland) using a ball-on-disc configuration. The tribometer has a friction force resolution of 0.06 mN [33,34]. This machine allowed for determining the wear resistance and the coefficient of friction of a pair of materials sliding relative to each other at the sliding speed and the applied load. The tests were performed under the following conditions:

- Load (P) = 10 N;
- Sliding velocity (V) = 0.1 m/s;
- Sliding distance (S) = 1000 m;
- Friction node: a ball of 100Cr6 steel;
- Lubricant-no;
- Radius: 12 mm.

**Figure 7:**
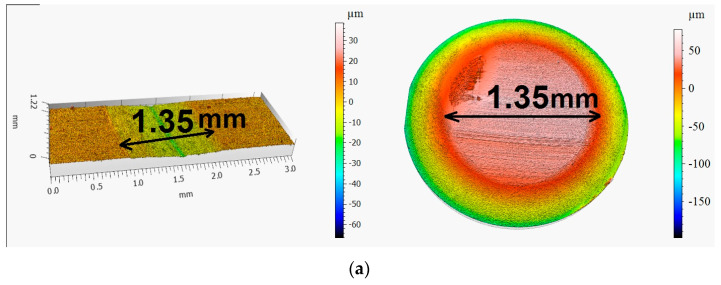

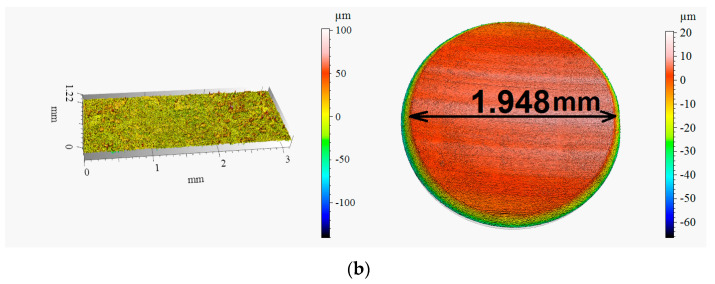
Isometric views of the discs and balls after the tribological tests at an ambient temperature. (**a**) visible signs of abrasion without the layer, and (**b**) sample covered with ESD, no signs of abrasion on the sample.

After the tribological tests, the surface geometry of the discs and balls was examined using a Leica DCM8 confocal microscope (Leica, Geneva, Switzerland) in interferometric mode. The microscope has an accuracy of <3% relative error (open loop) and <20 nm error (closed loop) at 20× magnification [33,35]. Figure 7 presents the isometric images and primary steel disc and ball profiles after wear tests.

### 2.6. Tomography Examination

The tomographic examinations were carried out on the NIKON M2 LES System device using computed tomography. The experiment used a microfocus source with a rotating tungsten anode. The rotating target had a maximum the accelerating voltage is set at 225 kV. The VAREX XRD 1611 XP detector (Varex Industrial, Salt Lake City, UT, USA)with a pixel size of 0.1 mm and a resolution of 4048 × 4048 pixels was used for archiving. The Inspect-X Version V6.8 (Nikon Metrology, Brighton, MI, USA) programme was used to process and transform 2D radiographic images into 3D tomographic reconstructions. Cross-section visualization and thickness measurements of the tungsten layer were performed in the VG STUDIO MAX version 2022.4 programme from Volume Graphics software (VG STUDIO MAX version 2022.4) [36].

The technique of testing using ionizing radiation involves archiving X-rays generated by an X-ray tube after passing through the tested object on the surface of a digital detector [36]. X-ray computed tomography creates images by detecting changes in the density of the material. This can reveal things like pores in castings or fragments of other elements inside the tested material [37]. Factors such as the material’s density and the decrease in radiation intensity can be used to predict the decrease in radiation reaching the detector [38].

**Figure 8:**
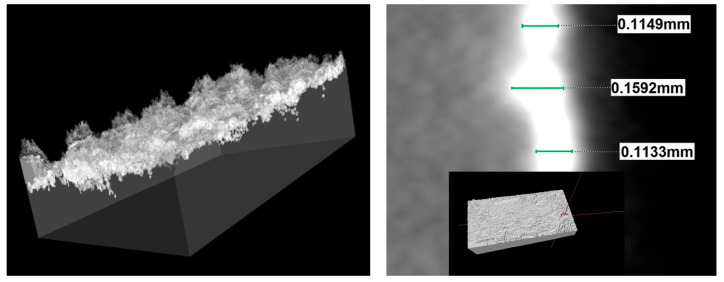
Three-dimensional image reconstruction of the investigated layer obtained by ESD technique.

Figure 8 displays a digitally “cut” fragment of the applied layer; a change in brightness corresponds to a change in the density of the material. Each layer that was received was tested and measured.

## 3. Discussion and Results

Based on the data obtained from the experimental tests, mathematical models of mass losses of the sample and counter-sample during the tests and the thickness of the formed layer were generated using Statistica software. Mathematical relations between the values of ESD process parameters and the values of layer thickness and component wear in friction nodes were possible thanks to the use of Response Surface Methodology (RSM) using an experimental design. The results were evaluated using analysis of variance (ANOVA). The ANOVA results for the adopted significance level α = 0.07 are presented in Table 3, Table 4, Table 5 and Table 6.

Multiple regression with backward elimination (for the adopted significance level α = 0.07) was used to describe the response function in the form of a second-degree polynomial. The homogeneity of variance was checked for a significance level of α = 0.07. Conclusions were drawn about the significance or insignificance of the influence of a specific term of the regression equation. Terms that were insignificant from the point of view of statistical inference were rejected, and only significant terms were taken into account [39,40,41,42].

The determined equations for the thickness of the layer and mass losses of the sample and counter-sample during tribological testing, as well as the correlation coefficients, determination coefficients, and corrected determination coefficients, are presented in Table 6.

The obtained mass change equations are characterized by a high degree of correlation, R, for mass losses and a high degree of correlation for layer thickness.

An analysis of the influence of individual input factors of the tungsten coating process on the variance of the Δ*mw*, Δ*mb* and *t* models indicates that the stress during the formation of layers using the ESD method has an impact on mass losses and layer thickness. The remaining terms and their mutual interactions (for the adopted significance level *α* = 0.07) are insignificant.

Based on the literature [43], the layer thickness obtained using ESD methods depends on the voltage and capacitance of the capacitors, relationship (1)

(1) $$Ep=\frac{1}{2}CU$$

where

*C*—capacitor capacity,

*U*—charging voltage.

The equations obtained in Table 6 show that the thickness of layers obtained by ESD methods and their operational properties (i.e., wear of the sample with coating I and the counter-sample) depend, for the assumed significance level *α* = 0.07, only on the voltage applied during coating application. It should be emphasized that the developed model applies only to coatings made of sintered tungsten on a grey cast iron substrate obtained using the available equipment. Further research will focus on analyzing the properties of coatings obtained from other metals. The authors pave the way for other scientists to conduct research on coatings obtained by ESD methods on other equipment.

**Figure 9:**
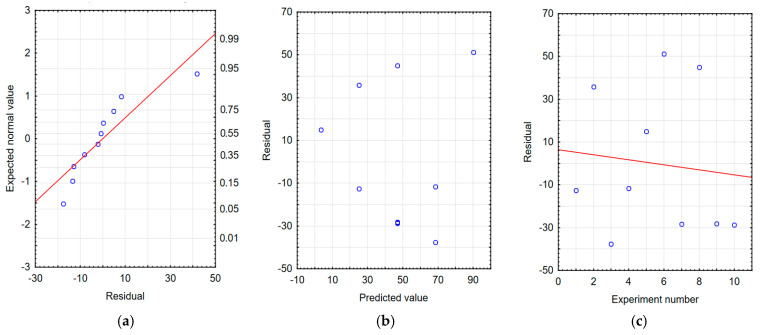
Residual analysis for the model of sample mass change after tribological testing of samples with tungsten coating. (**a**) Normal residual graph, (**b**) residuals relative to predicted values, and (**c**) residuals relative to the order of the experiment performed.

**Figure 10:**
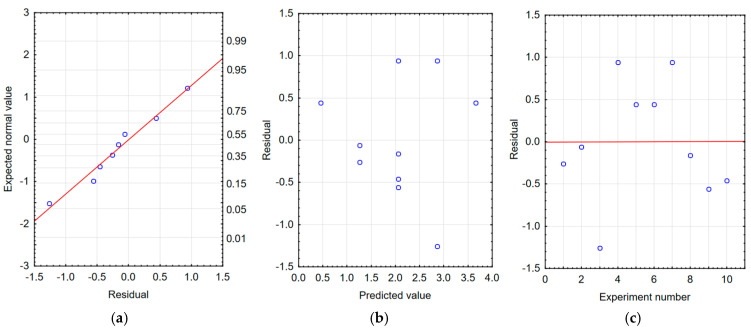
Residual analysis for the counter-sample mass change model: balls after tribological testing of samples with tungsten coating. (**a**) Normal residual graph, (**b**) residuals relative to predicted values, and (**c**) residuals relative to the order of the experiment.

**Figure 11:**
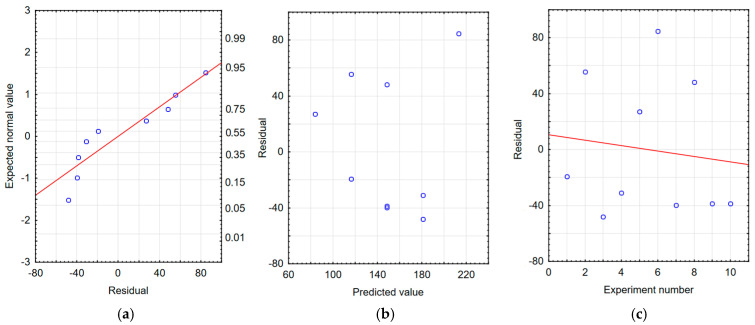
Residual analysis for the sample thickness model with tungsten coating. (**a**) Normal residual graph, (**b**) residuals relative to predicted values, and (**c**) residuals relative to the order of the experiment.

The correctness of the developed models was verified by residual analysis. Checking the graphs of the expected value versus the residuals, Figure 9a, Figure 10a and Figure 11a show a linear relationship, which indicates a good model of the function of the processing time and processing frequency. The dependence of the residuals on the predicted values shown in Figure 9b, Figure 10b and Figure 11b indicates the random nature of the residuals. Furthermore, based on Figure 9c, Figure 10c and Figure 11c, it can be concluded that the dependence of residues on the order of tests performed is random. The analyses confirm the adequacy of the developed regression models.

**Figure 12:**
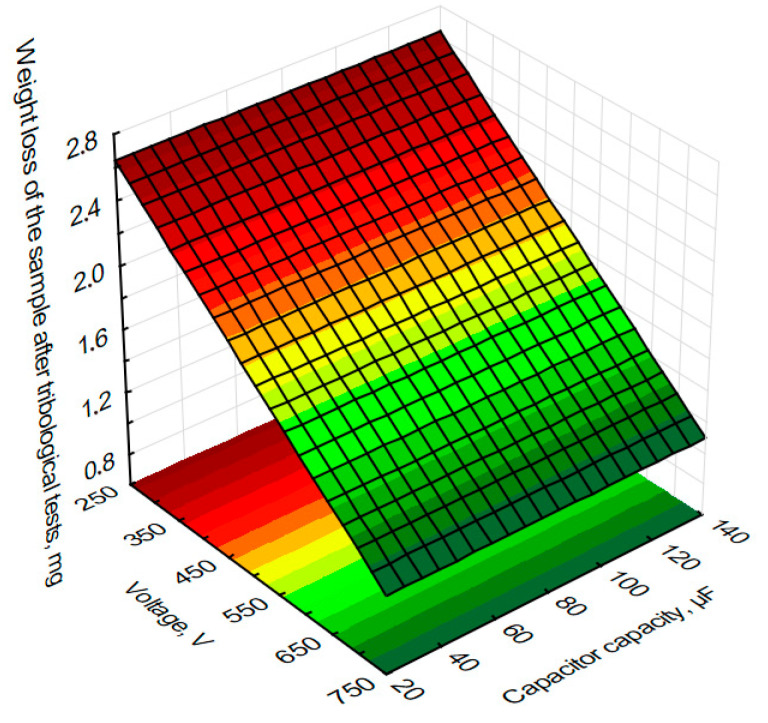
Estimated sample mass loss after tribological testing of voltage and capacitor capacity during the production of tungsten coatings using the ESD method.

**Figure 13:**
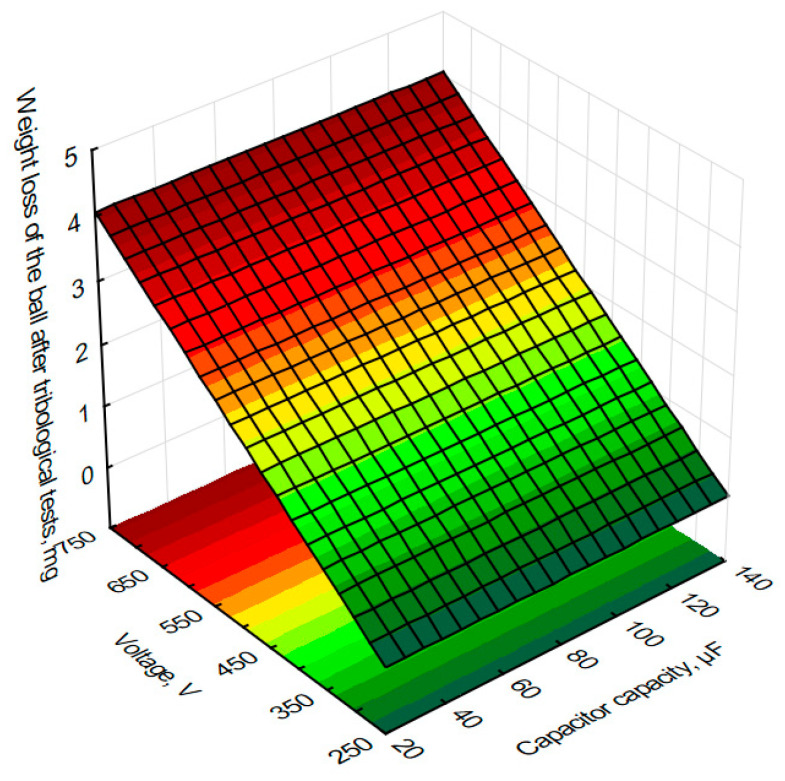
Estimated mass loss of the counter-sample—balls after tribological testing of voltage and capacitor capacity during the production of tungsten coatings.

**Figure 14:**
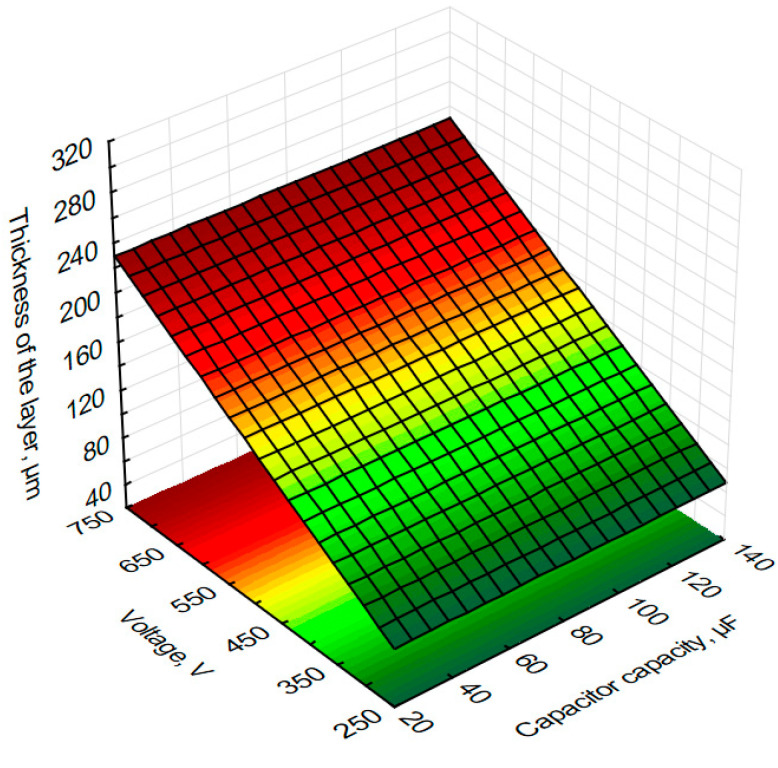
Estimated thickness of the tungsten layer from voltage and capacitance of capacitors during the production of ESD tungsten coatings.

In order to graphically illustrate the dependence of input factors on individual quality indicators (layer thickness) and performance indicators (mass loss of the sample and counter-sample—balls) of the ESD coating process, surface fits were used in the Statistica programme. This allowed us to prepare graphs showing the dependence of individual process indicators on capacitor capacity and voltage in Figure 12, Figure 13 and Figure 14.

In order to assess the effect of tungsten coating on friction conditions, a technically dry sample without coating was also tested. The sample without coating showed a weight loss of 7.5 mg. Based on the graph, it can be concluded that higher voltages result in lower weight losses of the sample during tribological testing. The greatest weight loss of 2.3 mg was observed for a voltage of 400 V and a capacitor capacity of 25 µF. The smallest sample mass loss of 0.9 mg was recorded for a capacitance of 700 V and a voltage of 75 µF. Based on experimental tests, an eightfold reduction in sample mass loss was obtained with a tungsten coating—0.9 mg—compared to analogous tribological tests of a sample without a coating—7.5 mg.

Based on the measurements and the graph of the mass loss of the counter-sample balls during tribological testing as a function of capacitor capacitance and voltage during the ESD tungsten coating process, it can be seen that the untreated sample is characterized by a ball mass loss of 0.3 mg. With a coating obtained at a capacitor capacitance of 25 µF and a voltage of 600 V, the ball weight loss increased 12-fold to 3.8 mg. The graph shows that the use of higher voltages increases the wear of the samples. The capacitance of the capacitors does not affect the ball weight loss at the assumed level of significance.

Based on the graph, the thickness of the layer as a function of voltage and capacitor capacitance during the production of tungsten coatings shows that the layer thickness increases with increasing ESD process voltage. The use of high voltages results in the formation of tungsten layers with greater thicknesses exceeding 200 µm. Experimental studies have shown that for a voltage of 700 V and a capacitor capacitance of 75 µF, a tungsten layer with an average thickness of 298 µm can be obtained. The smallest layer thicknesses of approximately 97 µm were obtained for a voltage of 400 V and a capacitor capacity of 25 µF. Based on the graph and the developed mathematical model, the parameters of the ESD coating process can be selected to obtain the desired layer thickness.

Characteristics of the coating layer: Figure 5 shows the SEM images of the cross-sections of the coating areas created in the ESD process. The ESD method is a type of microwelding process. The difference in the thermal expansion coefficients of the coating material and the base material may result in the top of the coating not being smooth.

**Figure 15:**
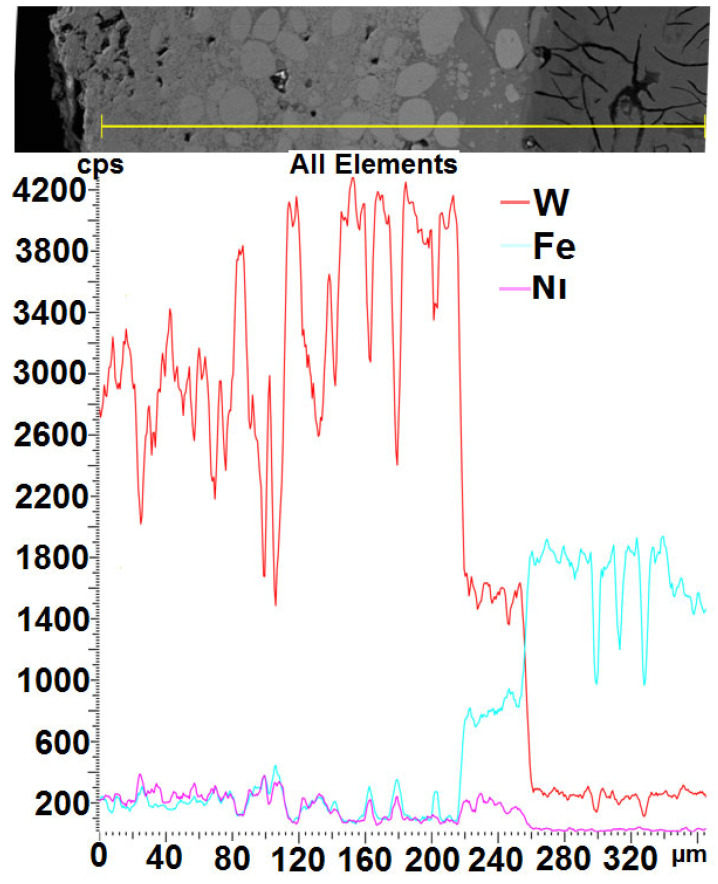
SEM analysis shows distribution of elements in coated layer of the mixing in the bonding zone (area of analysis: 220–260 µm).

W, Ni, and Fe are the main components of the shell. The different layer thicknesses are mainly due to the manual operation mode of the ESD gun used in this study, which has the disadvantage of uneven movement, speed, and pressure force during deposition, resulting in an uneven deposition process [44,45,46,47]. The abundance of Fe and W in the coating increased the hardness and thickness of the coating. Figure 8 shows that Fe, Ni, and W are included. The EDS shows a cross-sectional analysis of the crust. The EDS analysis of the crust is shown in Figure 15. A transient diffusion layer can be observed. W-Ni-Co in the coatings increases the microhardness of the samples. When W material is used together with elements such as Ni and Co, it can be easier to adhere to the substrate sample. Here, Ni and Co elements are used as binding materials. The use of bonding elements makes it difficult for cracks to form and spread within the coating and increases the hardness values of the coatings [48,49,50]. Such a linear distribution of elements allows you to hope for good adhesion of the layer. Diffusion does not occur at all points of the cross-section with the same intensity, which is caused by the pulsating nature of the layer formation process. The coating and the substrate do not completely mix due to the obvious difference in density, but there is some mixing in the bonding zone, as shown in Figure 15. The thickness of the coating was calculated to be 250 μm. In addition, due to the high solubility of nickel and the Fe-based substrate, many authors observed mixing of the coating and substrate during ESD treatment. Revealing the diffusion depth of individual areas of the observed layer is possible thanks to the use of X-ray microtomography.

The Vickers indentations show how the hardness of the layer changes in the zone of diffusion and combination of elements (Figure 6). The indications stabilize with the distance from the base material, showing only the hardness of the material applied with the ESD technique. These hardnesses do not differ from the hardness of the electrode material used for layering. These values vary from about 170–180 µHV for the base material, through the range of 510–530 µHV in the mixing and diffusion zone, to stabilize at slightly above 580–590 µHV in the ESD layer zone.

After the tribological tests, surface geometry was examined. Obtained layers provided the least wear, without a trace or with minimal wear tracks on the disc (Figure 7b), while the sample without the applied layer showed significant wear (Figure 7a). The opposite situation could be observed in the case of the ball, where the ball working with the material without the layer recorded lower wear than the ball working with the obtained layer.

Tomographic examinations confirmed the thickness of the layers obtained using the ESD method, which were measured using an optical microscope. Thanks to tomographic examinations, it is possible to successfully measure the obtained layers, especially when the substrate material and the deposited layer have such different densities. This does not require time-consuming cutting, embedding, polishing, and etching to observe the layer. The accuracy achieved with tomography using a standard reaches up to 6 µm.

In the present study, the electro-spark deposition (ESD) process was carried out manually. Hand-guided electrode manipulation inevitably introduces variations in the electrode travel speed across the substrate surface. Unlike automated systems, where electrode motion is stabilized and controlled, manual deposition depends strongly on the operator’s movement. This variability has several implications. First, “non-uniform coating thickness” is observed: slower electrode movement increases the local interaction time and energy input “microstructural inhomogeneity” may occur due to different degrees of substrate remelting and particle bonding, which in turn affects local hardness and tribological behaviour. Third, “irregular surface topography” may form in areas where excessive material accumulation occurs, resulting in micro-buildups and higher surface roughness. Finally, “process repeatability is limited”; manual ESD inherently produces greater variance in coating characteristics compared to automated deposition systems.

Consequently, manual ESD complicates the interpretation of experimental results. The fluctuating electrode speed obscures the isolated effect of technological parameters such as discharge current, pulse duration, and frequency on coating properties. Therefore, results obtained by the Authors, under manual conditions, are regarded as exploratory and representative of a laboratory-scale process.

## 4. Conclusions

The applied technique allows for the implementation of a coherent anti-wear layer on the selected substrate.

The material of the W-Ni electrode used allows for the production of a coherent and adhesive surface layer.

A diffusion bond between the layer and the substrate was observed.

The wide range of technical parameters obtained by the ESD device allows the use of a wide range of coating materials.

Initial tribological tests confirmed the assumptions about good anti-wear parameters of the obtained layers.

The application of tungsten coatings (using ESD technology) reduces wear on components in friction nodes. Experimental tests have shown an eightfold reduction in mass loss of samples with tungsten coatings compared to similar tribological tests on uncoated samples.

Experimental studies prove that an increase in voltage during the deposition of ESD tungsten coatings contributes to increased wear of counter-samples in friction joints. The use of appropriate ESD process parameters allows for changes in wear in friction joints and even allows for control of the degree of wear of individual friction joint components.

The developed mathematical model allows the selection of ESD coating process parameters to obtain the desired tungsten layer thickness. By using higher voltages, it is possible to obtain thicker tungsten layers, even up to 300 µm at a voltage of 700 V.

Manual execution of the ESD process introduces uncontrolled variability in electrode movement, which directly affects coating thickness, microstructure, and surface morphology. At the same time, manual deposition offers certain advantages, especially in exploratory and laboratory-scale studies. It allows for greater flexibility and adaptability, enabling the operator to adjust electrode positioning and contact pressure in real time, for example, when coating complex geometries or repairing localized defects. Moreover, manual ESD provides a cost-effective and rapid testing approach, useful for initial trials and proof-of-concept investigations without the need for expensive automation equipment. These features make manual ESD valuable for research and prototyping, despite its inherent variability.

In our opinion, considering the results, the best results were obtained for experiment No. 6 with input parameters of 75 µF and 700 V, which produced the thickest layer of approximately 300 µm and achieved the best abrasion resistance (wear).

## Figures and Tables

**Table 1:** Hartley’s five-level two-factor experiment plan.

No. Experiment	Capacitor Capacity—f, µF	Voltage—U, V	Δmw, Mass Loss of the Sample After Tribology Test, [g]	Δmb, Mass Loss of the Counter-Sample After Tribology Test, [g]	t, Average Thickness of the Layer, [µm]
1	25	400	0.0023	0.001	97
2	50	400	0.0021	0.0012	172
3	25	600	0.002	0.0016	133
4	50	600	0.0013	0.0038	150
5	75	300	0.0022	0.0009	111
6	75	700	0.0009	0.0041	298
7	50	500	0.002	0.003	109
8	133	500	0.0016	0.0019	197
9	75	500	0.0022	0.0015	110
10	75	500	0.0021	0.0016	110

**Table 2:** Chemical composition of the cast iron sample used in the experiment.

Fe—Cast Iron Composition					
C 3.8776	Si 3.9735	Mn 0.3373	P 0.0725	S 0.2386	Cr 0.0465
Mo < 0.002	Ni 0.0172	Al < 0.002	Cu 0.0367	B 0.0089	Ti 0.0516
V 0.0136	W < 0.01	Co 0.0074	Ca 0.0037	Nb 0.0027	Sn < 0.002
Pb < 0.005	Zn < 0.002	Zr 0.0045	Sb < 0.01	Ce < 0.005	La < 0.005
Mg 0.0012	Te 0.0004	Se < 0.003	Ta < 0.003	Fe 91.2984	-

**Table 3:** Results of ANOVA analysis for mass loss of the sample after tribological testing.

	Sum of Squares (SS)	Number of Degrees of Freedom	Mean Square	F	P	Influence %
Model	1.141	1	1.141	12.33	0.008	
*U*	1.141	1	1.141	12.33	0.008	100
Error	0.740	8	0.093			
Total SS	1.881	9	R^2^ = 0.61			R^2^-Adj = 0.56

**Table 4:** Results of ANOVA analysis of mass loss of counter-sample balls after tribological testing.

	Sum of Squares (SS)	Number of Degrees of Freedom	Mean Square	F	P	Influence %
Model	7.68	1	7.68	14.08	0.006	
*U*	7.68	1	7.68	14.08	0.006	100
Error	4.36	8	0.55			
Total SS	12.04	9	R^2^ = 0.64			R^2^-Adj = 0.59

**Table 5:** Results of ANOVA analysis for the thickness of the manufactured tungsten layer.

	Sum of Squares (SS)	Number of Degrees of Freedom	Mean Square	F	P	Influence %
Model	12,545.33	1	12,545.33	4.66	0.06	
*U*	12,545.33	1	12,545.33	4.66	0.06	100
Error	21,534.77	8	2691.85			
Total SS	34,080.10	9	R^2^ = 0.37			R^2^-Adj = 0.29

**Table 6:** Regression equations for the indicators of the tungsten layer production process.

The Regression Equation	R	R^2^	R^2^-Adj
∆mw=3.41−0.00308×U	0.78	0.61	0.56
∆mb=−1.94+0.008×U	0.80	0.64	0.59
t=−25.41+0.0896×U	0.59	0.35	0.27

## Data Availability

The original contributions presented in this study are included in the article. Further inquiries can be directed to the corresponding author.
